# Local delivery of USC-derived exosomes harboring ANGPTL3 enhances spinal cord functional recovery after injury by promoting angiogenesis

**DOI:** 10.1186/s13287-020-02078-8

**Published:** 2021-01-07

**Authors:** Yong Cao, Yan Xu, Chunyuan Chen, Hui Xie, Hongbin Lu, Jianzhong Hu

**Affiliations:** 1 Department of Spine Surgery and Orthopedics, Xiangya Hospital, Central South University, Changsha, 410008 China; 2 Key Laboratory of Organ Injury, Aging and Regenerative Medicine of Hunan Province, Changsha, 410008 China; 3 National Clinical Research Center for Geriatric Disorders, Xiangya Hospital, Central South University, Changsha, 410008 China; 4 Department of Sports Medicine, Xiangya Hospital, Central South University, Changsha, 410008 China; 5 Movement System Injury and Repair Research Center, Xiangya Hospital, Central South University, Changsha, 410008 Hunan China

**Keywords:** Spinal cord injury, Human urine stem cell, Exosome, Angiopoietin-like protein 3, Angiogenesis

## Abstract

**Background:**

Spinal cord injury is a devastating clinical condition for which there are currently no effective therapeutic options. In the present study, we aim to investigate if the effect of an administered injection of exosomes derived from human urine stem cell (USC-Exo) embedded in hydrogel could improve the spinal cord functional recovery after injury and the underlying mechanism.

**Methods:**

Exosomes were isolated from USC and identified by transmission electron microscopy (TEM) and Western blot. Functional assays in vitro were performed to assess the effects of USC-Exo on tube formation and migration, as well as their regulatory role in the PI3K/AKT signaling pathway activation. A locally administered injection of exosome embedded in hydrogel was used for SCI treatment. The effects of USC-Exo on functional recovery and the role of the candidate protein ANGPTL3 harboring in USC-Exo for promoting angiogenesis in SCI model were assessed.

**Results:**

In the current study, we demonstrate that a locally administered injection of USC-Exo embedded in hydrogel can pass the spinal cord blood-brain barrier and deliver ANGPTL3 to the injured spinal cord region. In addition, the administration of human USC-Exo could enhance spinal cord neurological functional recovery by promoting angiogenesis. The results of mechanistic studies revealed that ANGPTL3 is enriched in USC-Exo and is required for their ability to promote angiogenesis. Functional studies further confirmed that the effects of USC-Exo on angiogenesis are mediated by the PI3K/AKT signaling pathway.

**Conclusion:**

Collectively, our results indicate that USC-Exo serve as a crucial regulator of angiogenesis by delivering ANGPTL3 and may represent a promising novel therapeutic agent for SCI repair.

**Supplementary Information:**

The online version contains supplementary material available at 10.1186/s13287-020-02078-8.

## Background

Spinal cord injury (SCI) is a devastating clinical condition and a major cause of long-term disability. Patients surviving SCI are often left with paraplegia that significantly affects their quality of life [1]. Epidemiological data have demonstrated that the annual incidence of SCI is 15–40 cases per million and is rising worldwide [2]. China has a large number of SCI patients, with an estimated incidence of SCI of 60.6 per million in Beijing [3]. The financial burden associated with SCI is staggering, but despite major advances in the medical treatment for SCI in experimental animals, no fully restorative therapies currently exist for mammalian SCI.

SCI can produce an immediate disruption of the nervous tissue, with direct vessel injury leading to neuronal death [4]. Ruptured blood vessels in the injury epicenter cause hemorrhaging and inflammation, which contribute to the aggravation of SCI [4]. The insufficient formation of new functional vessels in and near the injury epicenter impedes endogenous neural tissue repair. Regenerating axons have been shown to grow along vessels [5], and abnormalities in vessel regeneration will delay tissue regeneration. The limited self-repair capability of the injured spinal cord underscores the need for regenerative interventions to restore the neurological function of SCI; thus, promoting post-injury angiogenesis can improve neurological function and may be a promising therapeutic target for SCI treatment.

Exosomes have attracted great attention in regenerative medicine in recent years due to their great potential to be used as therapeutic agents [6]. Exosomes transport molecules such as proteins and nucleic acids to target cells and regulate their biological processes [7]. Exosomes can be secreted from various cells, especially stem cells. Recently, accumulating evidence has shown that exosomes derived from stem cells mimic the phenotype of parent stem cells and can activate the self-regenerative programs of target cells, demonstrating their therapeutic potential for various diseases [8]. In our previous study, the systemic administration of mesenchymal stem cell (MSC)-derived exosomes promoted functional recovery and angiogenesis in animal models of SCI [9]. Human urine-derived stem cell (USC) can be harvested through a safe, simple, low-cost, and noninvasive method and is believed to be a promising stem cell candidate to obtain exosomes from. Studies have shown that exosomes derived from human USC can promote wound healing by enhancing angiogenesis [10]. However, the role of exosomes derived from human USC in SCI has not been explored. Interestingly, a proteomic analysis revealed that human USC-derived exosomes (USC-Exo) harbor multiple angiogenesis-related proteins, including ANGPTL3 [10]. ANGPTL3 is a liver-specific, secreted factor consisting of an N-terminal coiled-coil domain and a C-terminal FBN-like domain that facilitates regulating blood vessel formation [11]. ANGPTL3 is part of a family of angiogenic molecules, suggesting its potential role in regulating angiogenesis [11]. ANGPTL3 has been shown to exert a pro-angiogenic ability and promote cardiac angiogenesis after myocardial infarction [11], indicating that ANGPTL3 administration is a promising approach for SCI repair.

In the present study, we used a mouse SCI model to investigate the effect of USC-Exo following injury. Moreover, the specific role of the angiogenic molecule ANGPTL3 in USC-Exo cargo was assessed.

## Methods

### Isolation and identification of USC-Exo

Human urine stem cell-derived exosomes (USC-Exo) were isolated via ultracentrifugation as previously described [10]. Briefly, three healthy volunteers (man) with an age range of 20–30 years signed an informed consent to donate their urine samples (30–50 ml per sample) for the study; all experimental procedures were approved by the Ethical Review Board at Xiangya Hospital of Central South University (No. 201612653). After obtaining human USC, they were washed with PBS and cultured in exosome-free FBS (Thermo Fisher, USA) USC medium at 37 °C under an atmosphere with 5% CO_2_. Then, the conditioned medium was collected and centrifuged at 2000×*g* for 30 min. After centrifugation, the supernatant was filtered through a 0.22-μm filter (Millipore, Billerica, USA) to remove the cellular debris. Then, the supernatant was added to an ultraclear tube (Millipore, USA) and ultracentrifuged at 100,000×*g* twice, each for 2 h, to obtain exosome pellets. The USC-Exo pellets were resuspended in 200 μl of PBS and stored in a − 80 °C refrigerator for use in subsequent experiments. The USC-Exo fraction was examined and imaged with a transmission electron microscope (TEM; Hitachi H-7650, Hitachi, Japan). The size distribution and concentration of USC-Exo were determined by nanoparticle tracking analysis (NTA) using a NanoSight NS3000 system (NanoSight, Amesbury, UK) according to the manufacturer’s instructions. The USC-Exo protein contents were determined using a BCA™ Protein Assay kit (Thermo Fisher, USA). The specific exosome surface markers CD63, CD81, and TSG101 were identified by Western blot (WB) analysis.

### ANGPTL3 deletion using a shRNA plasmid

USCs were transfected using Lipofectamine LTX with Plus reagents (Invitrogen, Carlsbad, CA, USA). We established stable ANGPTL3 knockdown and control USC using ANGPTL3 shRNA (shANGPTL3) and control shRNA (shMock) vectors (Santa Cruz Biotechnology, Santa Cruz, CA, USA), respectively, according to the manufacturer’s protocol.

### USC-Exo internalization assays

USC-Exo were collected and labeled using a PKH26 red Fluorescent Cell Linker kit (Sigma Aldrich; Saint Louis, MO, USA) according to the manufacturer’s instructions. Then, the USC-Exo pellets were resuspended in 1 ml of Diluent C, after which 1 μl of PKH26 dye diluted in 250 μl of Diluent C was added and the USC-Exo were gently mixed for 4 min. Subsequently, an equal volume of 1% BSA was added to the reaction system to stop the reaction. Then, the PKH26-labeled exosomes were ultracentrifuged at 100,000×*g* for 70 min, washed with PBS, and ultracentrifuged again to remove excess dye. HUVECs were purchased from the Cell Bank of the Chinese Academy of Sciences (Shanghai, China). The PKH26-labeled USC-Exo were incubated with cultured human umbilical vein endothelial cells (HUVECs) and stained with 4′,6-diamidino-2-phenylindole (DAPI; Vectashield, Vector Laboratories, Burlingame, CA, USA) to visualize nuclear structures and were further analyzed with a fluorescence microscope (BIOREVO BZ7000; Keyence; Osaka, Japan).

### Tube formation assay

To investigate the effect of USC-Exo on HUVEC activity, the formation of capillary-like structures was assessed as previously reported [10]. Cold growth factor-reduced Matrigel (50 μl, BD Biosciences) was added to a 96-well plate. Then, HUVECs (2 × 10^4^ cells/well) were seeded onto the Matrigel and treated with medium in the presence or absence of USC-Exo under different conditions as follows: (1) HUVECs treated with the PBS group, (2) HUVECs treated with USC-Exo (50 μg/ml) from the shMock transfected USC groups, and (3) HUVECs treated with USC-Exo (50 μg/ml) from ANGPTL3-silenced human USC. After 12 h of treatment, tube formation was assessed using a bright-field microscope (BZ-8000, Keyence, Osaka, Japan). The ability to form capillary-like structures was quantified by determining the number of branch points and tubule lengths in five randomly chosen microscopic fields using ImageJ (National Institutes of Health).

### Cignal Finder reporter array

To determine what signaling pathways in HUVECs were affected by the USC-Exo treatment, a Cignal Finder Reporter Array plate (Qiagen, USA) assay was performed according to the manufacturer’s instructions. Briefly, attractene (0.6 μl/well) was distributed into a 96-well Cignal Finder Multi-Pathway Reporter Array plate. HUVECs were seeded in each well and incubated to allow complex formation. Then, the cells were treated with 200 μg of USC-Exo. Then, after 48 h of treatment, Dual-Glo Luciferase Reagent (75 μl) was added to the designated wells, and luciferase activity was measured according to the manufacturer’s recommendations. The luminescence signal was quantified by normalizing the ratios of cellular responses to the USC-Exo treatment (firefly luminescence signal) to the nonspecific responses to PBS (Renilla luminescence signal). The activities of the USC-Exo-treated cells were compared to those of the PBS-treated control cells, and the results were plotted as the log2-fold change. If log2 > 1, the difference was considered significant. To investigate whether PI3K/AKT signaling is involved in the USC-Exo-mediated effects on HUVECs, specific inhibitors of PI3K/AKT signaling (LY294002 and L-NAME) were used to assess their effects on tube formation.

### Western blot analysis

Proteins from cells or exosomes were extracted with RIPA buffer (R0278, Sigma). The protein concentration was determined using a Bradford microassay (Bio-Rad Laboratories, Hercules, CA), and proteins were separated by SDS-PAGE and ten transferred to iBlot nitrocellulose membranes (Invitrogen, Carlsbad, CA, USA). Membranes were blotted with primary antibodies against CD63 (Santa Cruz Biotechnology), CD81 (Santa Cruz Biotechnology), TSG101 (ProteinTech, Chicago, USA), ANGPTL3, GAPDH (Abcam, Cambridge, UK), PI3K, AKT, phosphorylated PI3K (p-PI3K), and AKT (p-AKT) (Cell Signal Technology). After washing with TBST, the membranes were incubated with appropriate HRP-conjugated secondary antibodies (Amersham Pharmacia). The immunoreactive bands were detected using enhanced chemiluminescence reagent (Thermo Fisher Scientific, Waltham, USA) and imaged with a ChemiDoc XRS Plus luminescent image analyzer (Bio-Rad).

### RNA isolation and quantitative reverse transcription PCR

Total RNA was extracted from the cultured cells and tissues using TRIzol Reagent (Invitrogen, Carlsbad, USA) and reverse transcribed into cDNA with a Revert Aid First Strand cDNA Synthesis kit (Fermentas, Burlington, Canada). RT-qPCR was performed using FastStart Universal SYBR Premix ExTaq (Takara Biotechnology, Japan) and analyzed on an ABI VII7 Real-Time RT-PCR system (Bio-Rad Laboratories Inc., Hercules, CA, USA). The sequences of primers used for RT-qPCR are listed in Supplementary Table 1. The fold changes of target mRNA expression relative to GAPDH were calculated based on the threshold cycle (*C*_T_) as *r* = 2^−Δ(ΔC^_T_), where Δ*C*_T_ = *C*_T_ (target) − C_T_ (*GAPDH*) and Δ (Δ*C*_T_) = ΔC_T_ (experimental) − ΔC_T_ (control).

### Mouse model of spinal cord contusion trauma and USC-Exo treatment

Animal experiment procedures were conducted according to the Guideline of Animal Care and Use Committee of Central South University (permit number: 20170101). Mice received spinal contusion trauma as previously described [12]. Briefly, after each mouse was fully anesthetized with 1 mg kg^−1^ of ketamine intramuscularly and 1.5–2.5% isoflurane, a vertebral laminectomy at thoracic T9–10 was performed to expose the spinal cord. A clinically relevant spinal cord contusion trauma was performed using a Horizons Impactor (Precision Scientific) (60 kdyn force) as previously described [13]. Mice were placed in a temperature- and humidity-controlled chamber, and manual bladder emptying was performed three times daily until reflex bladder emptying was established. The SCI mice were randomly divided into three groups (*n* = 8/group) and treated with 200 μl of PBS containing PKH26, 200 μg of exosomes labeled with PKH26 from shMock-transfected human USC in 200 μl of PBS, or 200 μg of exosomes labeled with PKH26 from ANGPTL3-silenced USC in 200 μl of PBS embedded in hydrogel and administered post-SCI by local intrathecal injection. The functional recovery from spinal cord injury was measured at different time points through locomotor behavioral assessments as well as electrophysiological, morphological, and immunofluorescence analyses and 3D vessel assessment.

### In vivo imaging of DiR-labeled USC-Exo in spinal cord injury models

To track USC-Exo in vivo, we labeled USC-Exo with 1 μM fluorescent lipophilic tracer DiR (Invitrogen, Life Technologies) as previously described [14] and embedded them in hydrogels, which was then transplanted to the surface of the injured spinal cord area. Animals were euthanized and placed in a Xenogen IVIS Imaging System (Caliper Life Sciences) to detect red fluorescence for biodistribution analysis.

### Locomotor functional recovery assessment

Mouse locomotor function was evaluated at 1, 3, 7, 14, 21, 28, and 56 days post-SCI by two examiners who were blinded to the experimental design according to the Basso Mouse Scale (BMS) system [15]. The BMS is a 10-point locomotor rating scale ranging from 0 (complete hindlimb paralysis) to 9 (normal locomotion) points. Briefly, mice were allowed to walk in an open field, and the examiners took notes of their ankle movement, plantar placement, weight support, stepping, coordination, paw position, and trunk stability. The mean score of both examiners for the two hindlimbs was used as the BMS score for each mouse.

### HE staining and cavity analysis

The mice were sacrificed at 28 days after SCI (*n* = 8/group), and the injured spinal cords were carefully removed, fixed in 4% paraformaldehyde, and embedded in paraffin after dehydration. Eight 5-μm-thick transverse sections 100 μm from the injury epicenter of the spinal cord were generated and stained with HE to assess the tissue morphology and identify the cavity site. The identified lesion areas in each section were measured using ImageJ (National Institutes of Health).

### Motor-evoked potential (MEP) recoding

The MEPs of the hindlimb were assessed by electromyography at 8 weeks post-surgery as previously described [16]. After effective anesthesia, the stimulating electrodes were secured onto the surface of the skull corresponding to the motor cortex area, and recording electrodes were inserted into the tibialis anterior muscle in the contralateral hindlimb. The reference electrodes were inserted into subcutaneous tissue between the stimulating and recording electrodes to obtain recordings in the target muscles. Mean MEP values, including latency and amplitude period, were recorded. The latency period was measured as the length of time from the stimulus to the onset of the first response wave. The amplitude period was measured from the initiation point of the first response wave to its peak point.

### Immunofluorescence analysis

For immunofluorescence staining, the sections were rehydrated and blocked with 5% BSA and then incubated with primary antibodies against Ki67 (Abcam) or anti-CD31 (Abcam). Then, the sections were incubated with the appropriate Alexa Fluor 488 goat anti-mouse IgG (H + L) antibody and Alexa Fluor 594 horse anti-rabbit secondary antibody. A DAPI solution was used for nuclear staining. Images were examined under a fluorescence microscope (Leica). Vessel density and Ki67/CD31 double-positive cells were quantified from five randomly selected visual fields per section using ImageJ (National Institutes of Health).

### 3D vessel analysis using synchrotron radiation micro-CT

The effects of USC-Exo on angiogenesis in rats were assessed using a novel synchrotron radiation micro-CT (SRμCT) approach at the BL13W beamline in the Shanghai Synchrotron Radiation Facility (SSRF). The imaging for SRμCT scanning was set up according to the previously described methods. Briefly, animals at 28 days post-SCI were anesthetized. Then, heparinized saline was perfused into the circulatory system to allow for effective blood drainage, which was followed by 10% buffered formalin perfusion for vessel fixation. The mixed contrast agent Microfil (Flow Tech, CA) was infused into the spinal cord microvasculature system via a perfusion pump as previously described [17]. A 5-mm-long spinal cord segment at the T10 thoracic cord that included the injury site was harvested and prepared for SRμCT scanning. After image acquisition, the ImageJ 3D Skeletonization plugin was used to extract the vascular 3D skeleton. The morphological parameters of the vascular network, including vessel volume fraction (VVF), vessel segment number (VSN), and vessel bifurcation number (VBN), were calculated using Image-Pro Analyzer 3D (Version 7.0; Media Cybernetics, Rockville, MD, USA) according to previously reported methods [17].

### Statistical analysis

The results were statistically analyzed with SPSS 22.0 (SPSS, Inc.). All data are presented as the means ± standard deviation (SD). Statistical analysis of multiple-group comparisons was performed by one-way analysis of variance (ANOVA), followed by the Bonferroni post hoc test. Values of *p* less than 0.05 were considered statistically significant.

## Results

### Characterization of USC-Exo

**Fig. 1:**
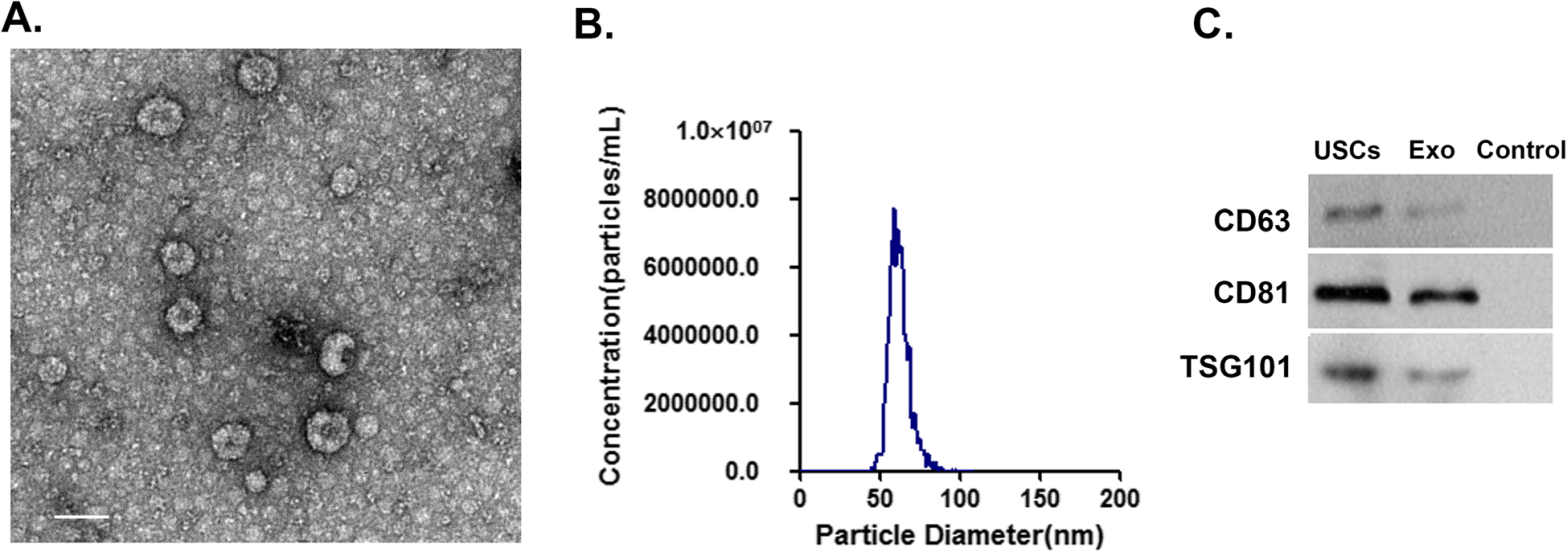
Characterization of isolated USC-Exo. **a** Representative images of USC-Exo morphology detected by transmission electron microscopy (TEM). Scale bar, 100 nm. **b** Size distribution assessed by nanoparticle tracking analysis (NTA) using a NanoSight NS3000 system. **c** Western blot analysis of specific exosomal surface markers

Isolated USC-Exo were examined by TEM, which showed the presence of rounded particles with a membrane-like bilayer (Fig. 1a). A NanoSight NS3000 instrument was used to determine the size distribution of the USC-Exo, which ranged from 40 to 100 nm in diameter (Fig. 1b). Western blot results demonstrated that the USC-Exo expressed the classical exosomal markers CD63, CD81, and TSG101. These data indicated that the extracellular vesicles derived from USC were predominantly exosomes.

### USC-Exo promote neurological functional recovery after SCI

**Fig. 2:**
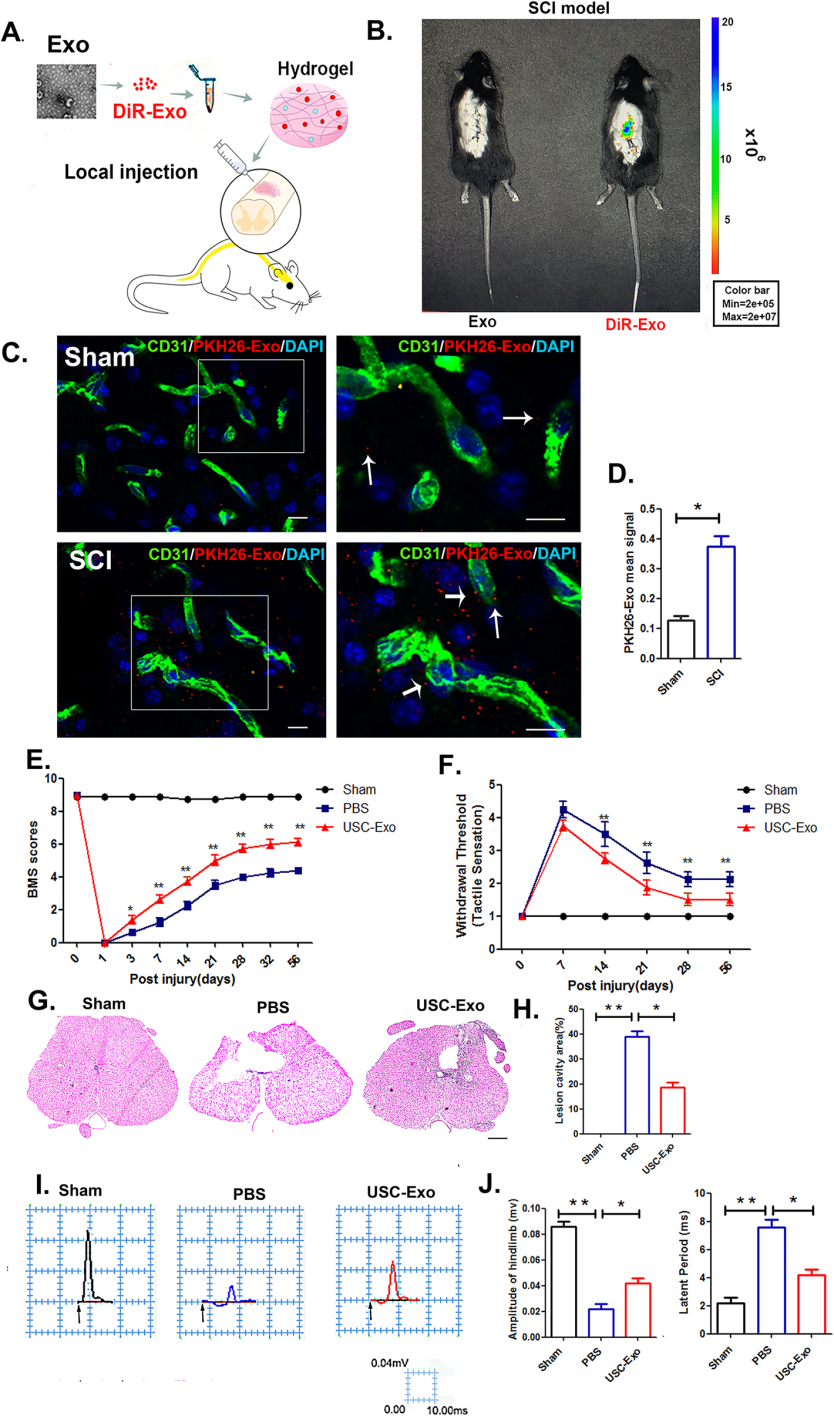
Locally delivered labeled USC-Exo home to the injured spinal cord site and promote locomotor and sensory function recovery. **a** Schematic representation of the procedure used to administer labeled USC-Exo. **b** In vivo imaging of animals that received a local intrathecal injection with/without DiR-labeled USC-Exo embedded in hydrogels in mice. **c** Immunofluorescence staining of the T10 spinal segment area in the sham (upper panel) and injured (lower panel) mice. CD31 (green), PKH26-Exo (red), nuclei (blue). Scale bar = 10 μm. **d** Quantitative PKH26-Exo mean signal intensity in the endothelial cells of the T10 spinal segment in sham and injured mice. **e** Distribution of the BMS scores per group throughout the 56-day period. **f** Distribution of sensory recovery per group at 0, 7, 14, 21, 28, and 56 days post-USC-Exo treatment. **g** Representative transverse HE stained sections 56 days post-SCI at distances 200 μm caudal to the injury epicenter. Scale bar = 1 mm. **h** Graphical representation showing the quantitative data comparing lesion cavities among different treatment groups. **i** Representative electrophysiological traces per group at 56 days post-SCI. **j** Quantification of the amplitude and latent period of MEPs in the USC-Exo-treated, PBS-treated, and sham mice. Two-way ANOVA with Tukey’s multiple comparisons. The data are presented as the means ± SD; *n* = 6 per group. *p < 0.05, **p < 0.01 compared with different treatment groups

After characterizing the USC-Exo, we next evaluated their therapeutic effects on neurological functional recovery after SCI. We traced the distribution of the DiR-labeled exosomes embedded in hydrogel after local treatment in vivo (Fig. 2a) using the Xenogen IVIS Imaging System, and the results demonstrated that DiR-exosome fluorescence accumulated in the injury area of the spinal cord. In contrast, no fluorescence signal was detected in the control mice where the free DiR-labeled exosomes embedded in hydrogel were injected (Fig. 2b).

For in vivo tracing of the uptake of PKH26-labeled exosomes by spinal cord vascular endothelial cells, ex vivo spinal cord slice imaging was performed and revealed that the PKH26-labeled exosomes could cross the blood-brain barrier (BBB) and be delivered to the injury sites of the spinal cord, where they were internalized by vascular endothelial cells (Fig. 2c). However, a weaker signal was observed in the sham spinal cord group (Fig. 2d). The expression of regeneration-associated genes (RAGs; ChAT, Hoxd10, and Lhx5) following USC-Exo administration in the injured spinal cord tissue was upregulated relative to that observed in the control groups at 7 days post-injury, indicating that the USC-Exo could promote spinal cord regeneration (Supplementary Figure 1). The functional analysis, as shown in Fig. 2e, showed that all animals exhibited no hindlimb movement at day 1 after SCI (BMS; 0). However, in agreement with the gene expression data, the motor function test results after mice were treated with USC-Exo showed substantially improved locomotor recovery after SCI, as demonstrated by increased BMS scores starting at 7 days post-injury until 56 days after SCI. In addition, the withdraw threshold responses to mechanical stimuli significantly decreased in the USC-Exo-treated mice over time post-SCI compared to the PBS-treated mice (Fig. 2f), indicating sensory improvement in the USC-Exo-treated mice post-SCI. We assessed whether the improved motor skills in the USC-Exo-treated mice were associated with reduced tissue injury after SCI. Histological sections stained with HE at distances of 200 μm caudal to the injury epicenter demonstrated decreased lesion in the USC-Exo-treated mice compared to that observed in the PBS-treated mice at 56 days after injury (Fig. 2g, h). Moreover, electrophysiological analysis revealed that the amplitude of motor-evoked potentials (MEPs) was significantly increased in the USC-Exo-treated mice compared to the PBS-treated mice at 56 days post-SCI. However, the latent period was downregulated in the USC-Exo-treated groups, further demonstrating the protective effect of USC-Exo against functional loss in SCI (Fig. 2i, j).

### USC-Exo promote angiogenesis after SCI

**Fig. 3:**
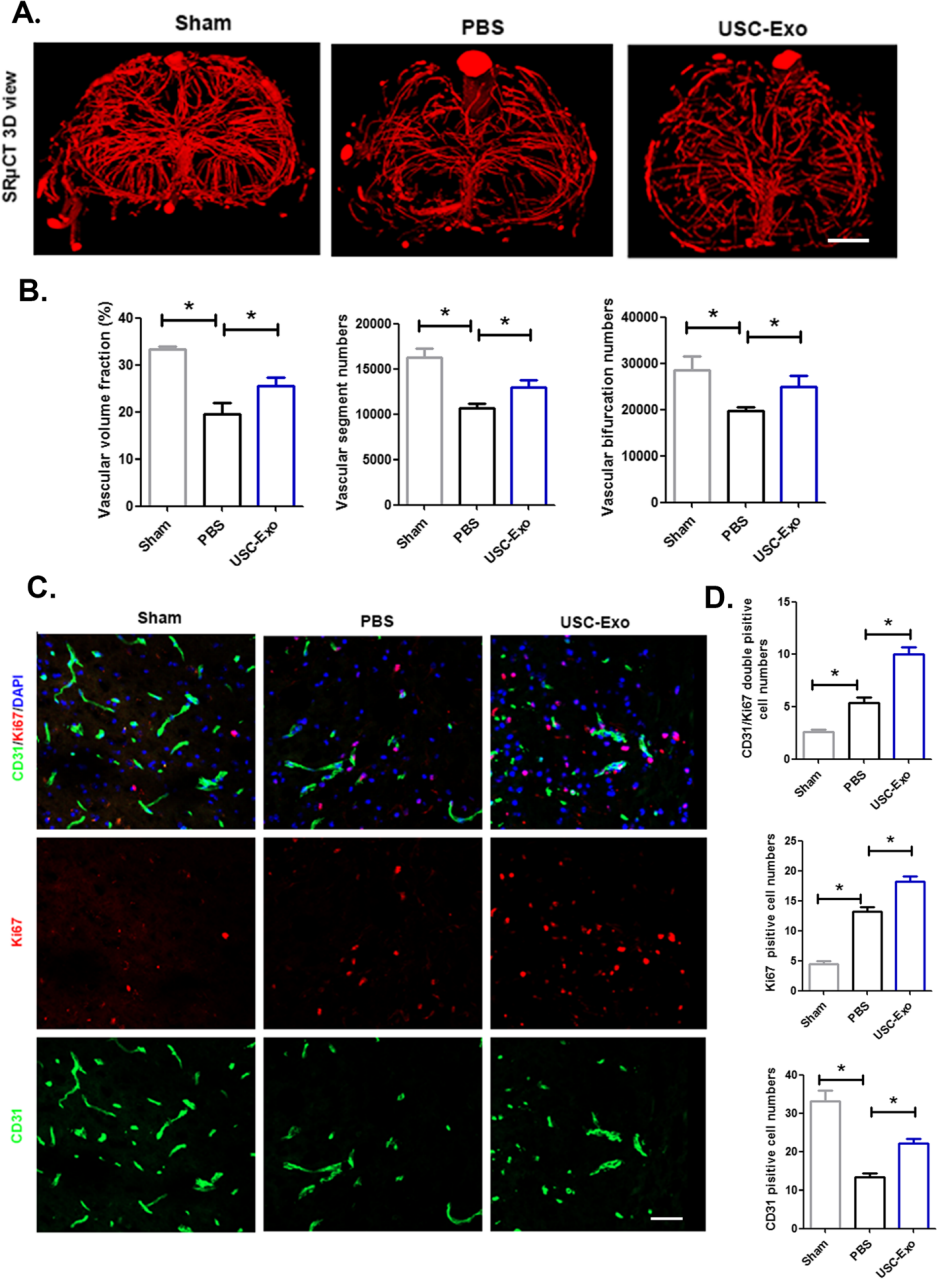
The administration of USC-Exo promotes angiogenesis after SCI. **a** Representative 3D image of spinal cord microvasculature at the T10 level per group at 56 days post-SCI visualized by SRμCT. Scale bars = 100 mm. **b** Quantification of the vascular volume fraction, vascular segment, and bifurcation numbers of the spinal cord among different treatment groups. **c** Representative immunofluorescence images of CD31 (green) and Ki67 (red) blood vessels in the mouse spinal cord in each group at 7 days post-SCI; scale bars = 50 μm. **d** Quantification of CD31 Ki67 double-positive cells in the spinal cords of mice among the different treatment groups. The data are presented as the means ± SD; *n* = 6 per group. *p < 0.05, **p < 0.01 compared among different treatment groups

To elucidate the underlying mechanism associated with the effect of USC-Exo on neurological function recovery after SCI, we conducted angiogenesis assays both in vivo and in vitro. The 3D microvasculature of the spinal cord in the USC-Exo-treated mice and PBS-treated mice at 56 days post-SCI was systematically analyzed using synchrotron radiation micro-CT (SRμCT) (Fig. 3a). The quantitative analysis of spinal cord microvasculature morphological parameters demonstrated that the USC-Exo could promote functional vessel regeneration after SCI compared to the PBS treatment groups, as indicated by the increased vessel volume fraction, vessel segment, and bifurcation numbers of the spinal cord in the USC-Exo-treated mice compared to that observed in the PBS-treated mice (Fig. 3b). In addition, immunostaining of spinal cord sections demonstrated that the number of Ki67 CD31double-positive cells significantly increased at 7 days post-SCI in the USC-Exo treatment groups compared to that observed in their age-matched PBS treatment groups, indicating that USC-Exo could increase angiogenesis post-SCI and was associated with improved neurological functional recovery (Fig. 3c, d).

### USC-Exo promote the angiogenic activity of HUVECs

**Fig. 4:**
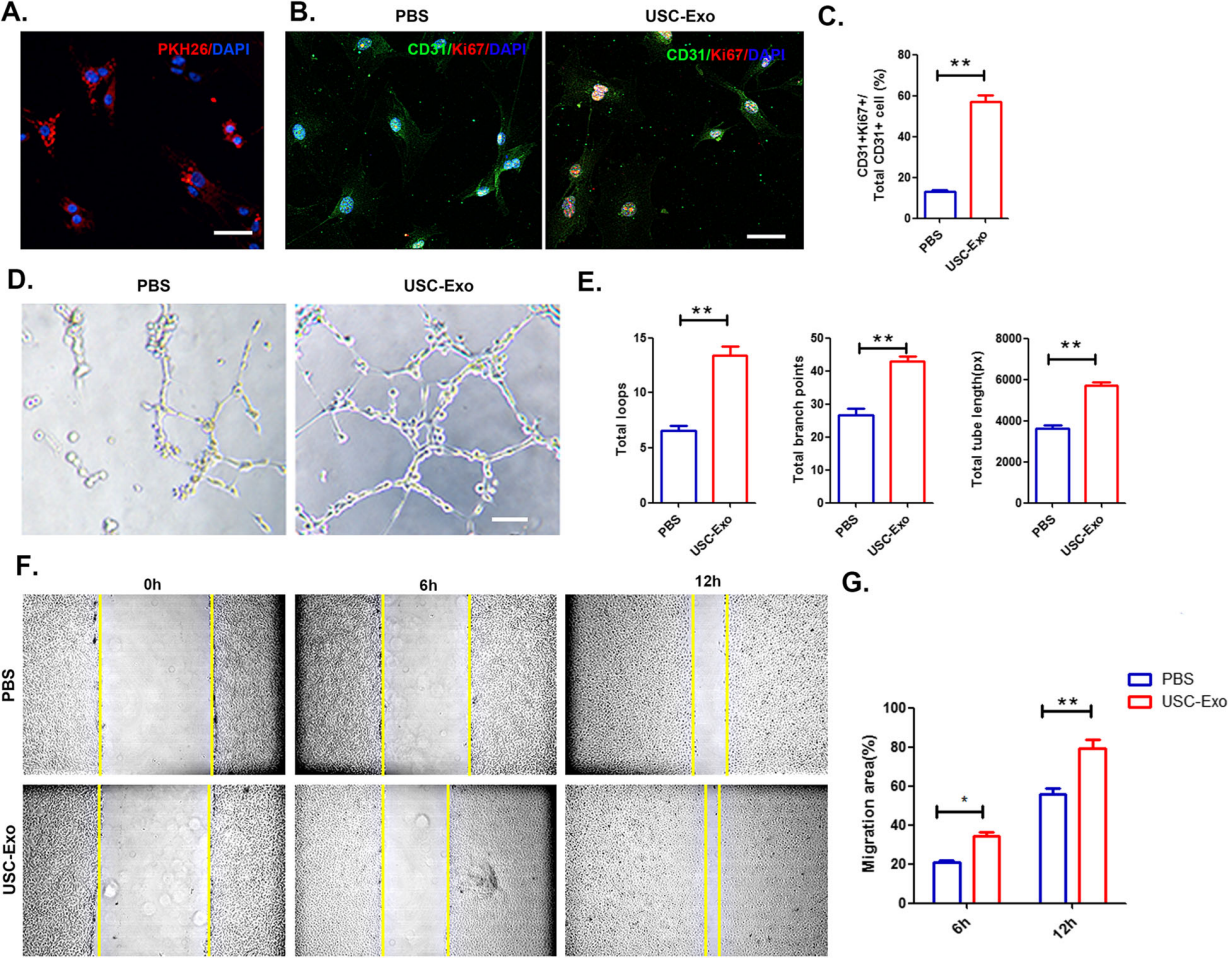
Internalization of USC-Exo by HUVECs promotes their angiogenic activities. **a** PKH26-labeled USC-Exo uptake by HUVECs was assessed by confocal microscopy. Scale bar, 20 μm. **b** Representative image of the Ki67 staining results after USC-Exo stimulation. Proliferative cells were stained red with Ki67. Scale bar, 20 μm. **c** After cultivation with USC-Exo, the number of CD31 Ki67 double-positive cells significantly increased. **d** Representative images of HUVEC tube formation in vitro after USC-Exo treatment. **e** Quantitative evaluation of the number of total loops, branch points, and total tube length after treating HUVECs with USC-Exo. **f** Representative images of HUVEC migration in the PBS and USC-Exo treatment groups in the scratch assay. **g** The percentage of migration area calculated for the USC-Exo or PBS treatment groups shown in **f**. The data are presented as the means ± SD; *n* = 6 per group. *p < 0.05, **p < 0.01 compared among different treatment groups

To examine whether USC-Exo could regulate the angiogenic activity of endothelial cells, we incubated HUVECs with USC-Exo for 24 h. We observed that after treatment with USC-Exo, the labeled exosomes could be taken up by endothelial cells and visualized by confocal microscopy (Fig. 4a). The Ki67 test showed that the proliferation of HUVECs significantly increased after USC-Exo stimulation (Fig. 4b, c). Additionally, USC-Exo induced more tube formation than that observed in the PBS treatment groups (Fig. 4d). As shown in Fig. 2e, after incubating for 24 h with USC-Exo, there was a significant increase in total loops (from 6.6 ± 0.8 to 14.2 ± 1.2), total branching points (from 26.6 ± 4.4 to 42.8 ± 3.3), and total tube length (from 3619 ± 360.6 to 5726 ± 280.4). A scratch wound assay was used to evaluate the effects of USC-Exo on cell mobility in HUVECs. Compared to the PBS-treated cells, USC-Exo-treated HUVECs exhibited a remarkable increase in cell migration (Fig. 4f, g). Taken together, these data indicated that USC-Exo have an angiogenic effect on HUVECs.

### USC-Exo affect the angiogenic activity of HUVECs by modulating the PI3K-Akt signaling pathway

**Fig. 5:**
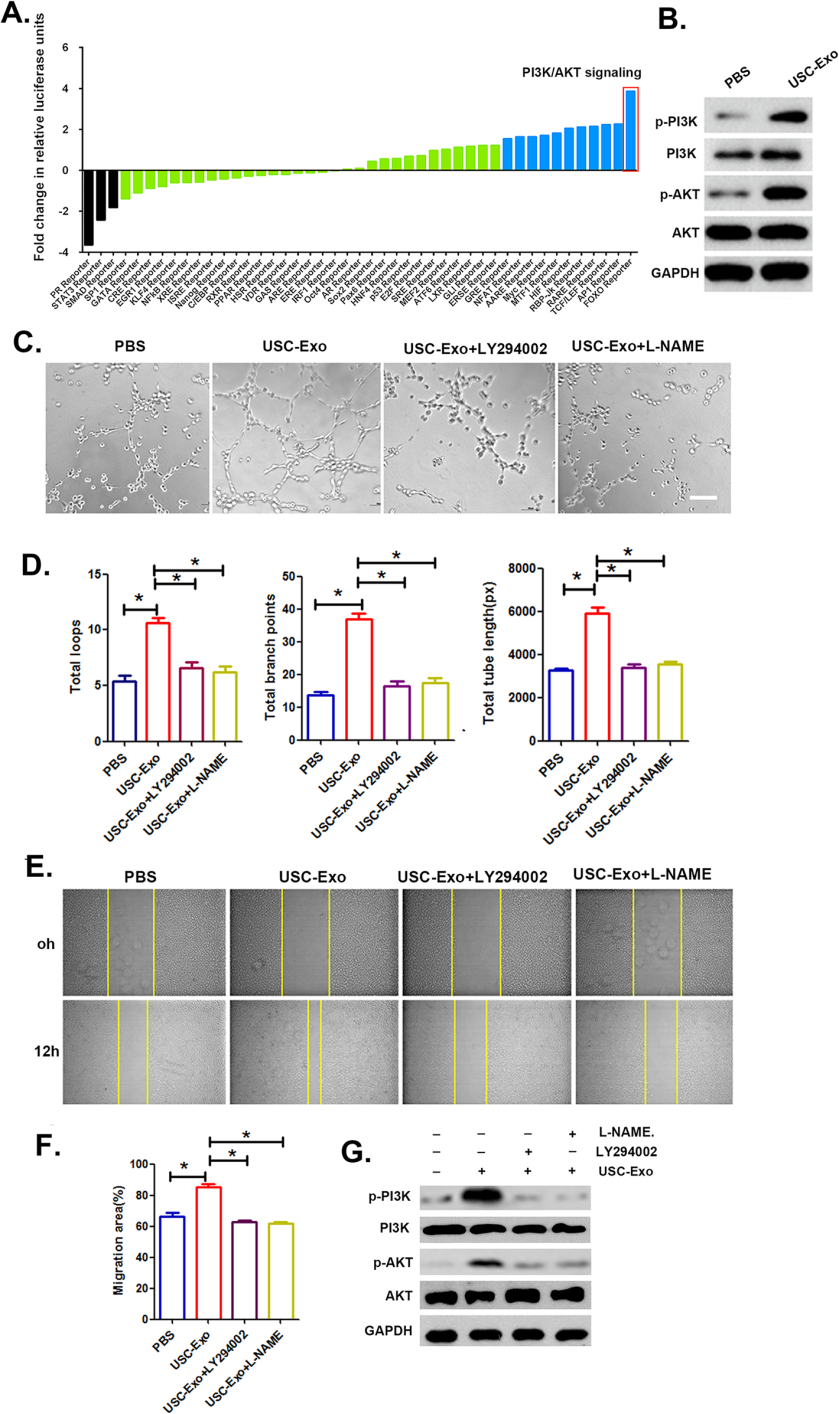
Comparison of 45 different signal transduction pathways following USC-Exo treatment in HUVECs. **a** Pathway reporters are listed by log2 ratio fold-change values in HUVECs treated with USC-Exo compared to cells treated with PBS. The results revealed that the PI3K/AKT signaling cascade changed the most in HUVECs after treatment with USC-Exo. **b** The expression of PI3K/AKT signaling pathway components in HUVECs after treatment with USC-Exo was detected by Western blot analysis. **c** Representative images of HUVEC tube formation in vitro after USC-Exo treatment with added LY294002 and L-NAME. **d** Quantitative analysis of the number of total loops, branch points, and total tube length shown in **c**. **e** Representative images of HUVEC migration in the USC-Exo treatment groups with added LY294002 and L-NAME in the scratch assay. **f** The percentage of the migration area calculated from **e**. **g** Western blot analysis of phosphorylated and total PI3K/AKT levels in HUVECs upon combination LY294002 and L-NAME treatment. The data are presented as the means ± SD; *n* = 6 per group. *p < 0.05, **p < 0.01 compared among different treatment groups

To elucidate the underlying molecular signaling pathways activated in HUVECs following USC-Exo stimulation, a Cignal™ 45-Pathway Reporter Array was used to assess signaling pathway activation, and the results revealed that PI3K/AKT signaling exhibited the greatest fold changes due to the administration of USC-Exo (Fig. 5a). We further performed a Western blot (WB) assay and observed that the protein levels of p-AKT and p-PI3K were significantly increased in HUVECs following USC-Exo treatment (Fig. 5b). To investigate how PI3K/AKT pathway activation mediated by USC-Exo exerts angiogenic effects on HUVECs, we examined HUVECs treated with USC-Exo with or without LY294002 or L-NAME. The USC-Exo-treated HUVECs showed significantly higher tube formation and cell mobility than the control group, whereas these effects were blocked by the addition of the PI3K inhibitor LY294002 or the AKT inhibitor L-NAME (Fig. 5c, e). Quantitative analysis of the total tube length, total branching points, total loops, and migration area further confirmed that the treatment of HUVECs with LY294002 or L-NAME blocked USC-Exo positive effects on tube formation and cell migration (Fig. 5d, f). In addition, the increased levels of p-AKT and p-PI3K in HUVECs resulting from USC-Exo treatment were significantly impaired after HUVECs were cultured with the PI3K inhibitor LY294002 or the AKT inhibitor L-NAME (Fig. 5g). These results indicated that the angiogenic effect of USC-Exo on HUVECs relies on PI3K/AKT pathway activation.

### USC-Exo transfer ANGPTL3 to HUVECs and mediate angiogenic activity

**Fig. 6:**
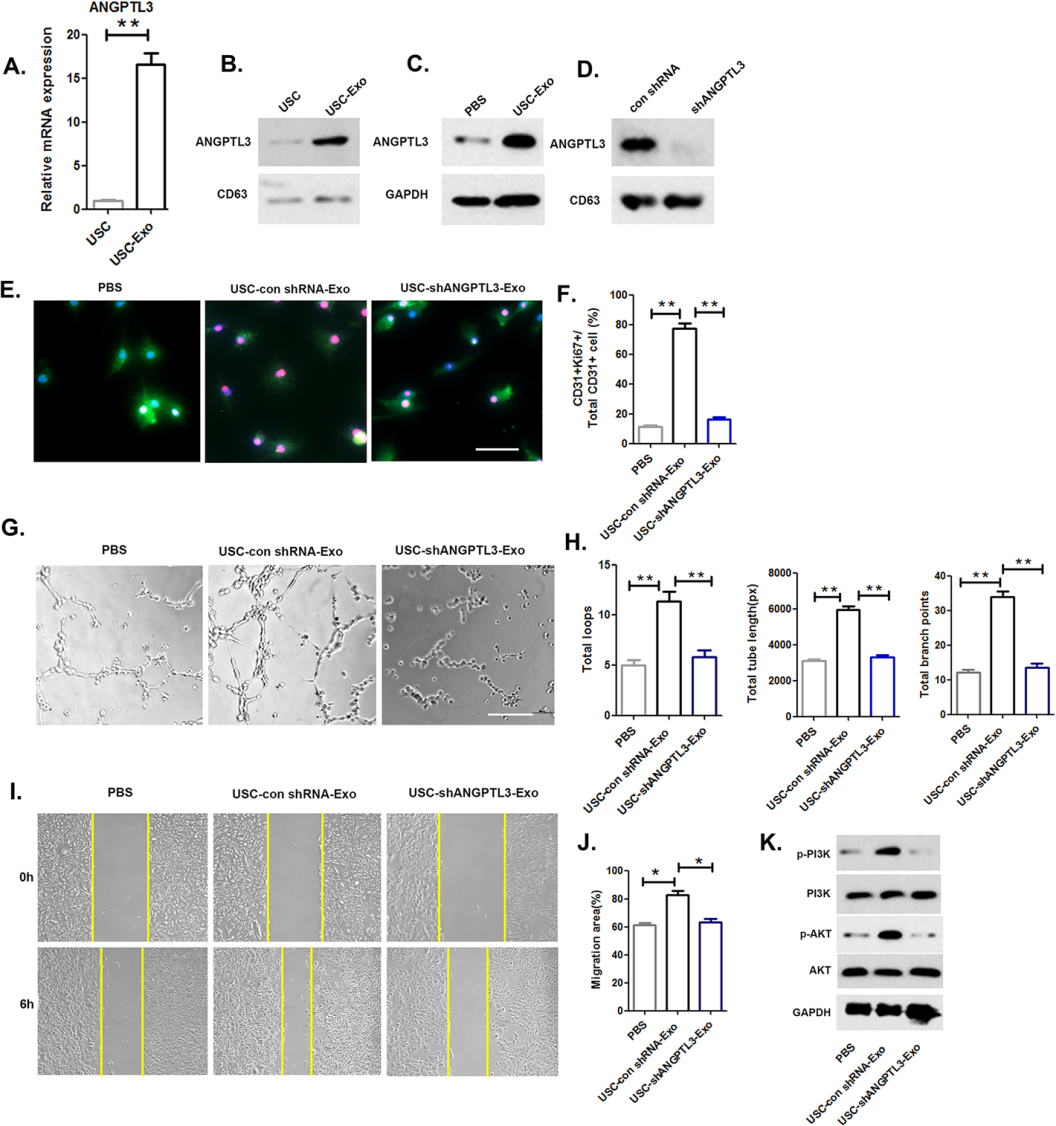
ANGPTL3 mediates the angiogenic effects of USC-Exo on HUVECs. **a**, **b** The mRNA and protein levels of ANGPTL3 were assessed by RT-qPCR and Western blot analysis. **c** Western blot analysis of ANGPTL3 in HUVECs after USC-Exo treatment. **d** Western blot analysis of ANGPTL3 in exosomes from ANGPTL3-silenced USC and confirmation of the inhibitory efficiency of shRNA targeting ANGPTL3. **e** The proliferation of HUVECs treated with PBS, USC-Con shRNA-Exo, and USC-shANGPTL3-Exo. **f** Quantitative analysis of the proliferation of HUVECs in different treatment groups shown in **e**. **g**, **i** Representative images of tube formation and migration in HUVECs treated with PBS, USC-Con shRNA-Exo, and USC-shANGPTL3-Exo. **h**, **j** Quantitative analyses of the total tube length, total branching points, and total loops shown in **g** and migration area shown in **i**. **k** Western blot analysis of the protein expression levels of PI3K/AKT components to assess signaling pathway activation in HUVECs among the different treatment groups. The data are presented as the means ± SD; *n* = 6 per group. *p < 0.05, **p < 0.01 compared among different treatment groups

We assessed the levels of angiogenesis-related proteins and observed that ANGPTL3 was enriched in USC-Exo, and quantitative analysis confirmed that the mRNA level of ANGPTL3 within USC-Exo was 16.6 ± 2.01-fold higher than that observed in USC (Fig. 6a). The protein level of ANGPTL3 in USC-Exo was relatively higher than that observed in USC (Fig. 6b). In addition, following treatment with USC-Exo, the protein level of ANGPTL3 in recipient HUVECs was upregulated compared to that observed in the control (Fig. 6c). We then investigated the role of ANGPTL3 in USC-Exo-mediated angiogenic activity in HUVECs using shRNA to knockdown ANGPTL3 expression in USC, and the level of ANGPTL3 was decreased in USC-Exo (Fig. 6d). The proliferation assay results demonstrated a decreased ability of USC-Exo to promote the proliferation of HUVECs when ANGPTL3 was knocked down in USCs (Fig. 6e, f). These data revealed that the pro-angiogenic activity of USC-Exo on HUVECs was suppressed by ANGPTL3 attenuation. The tube formation assay results demonstrated that fewer capillary-like structures formed when cells were treated with ANGPTL3-downregulated USC-Exo compared to that observed in the USC Con shRNA-Exo group (Fig. 6g, h). As demonstrated by the scratch assay results, the promigratory effects of USC-Exo were also suppressed once ANGPTL3 was downregulated in USC-Exo (Fig. 6i, j). Additionally, we conducted Western blot analyses to assess PI3K/AKT signaling pathway activation in HUVECs following treatment with USC-shANGPTL3-Exo, USC-Con shRNA-Exo, or an equal volume of PBS. As shown in Fig. 6k, PI3K/AKT signaling activation was markedly attenuated when ANGPTL3 expression in USC-Exo was inhibited.

### ANGPTL3 mediates the protective effect of USC-Exo on neurological functional recovery after SCI

**Fig. 7:**
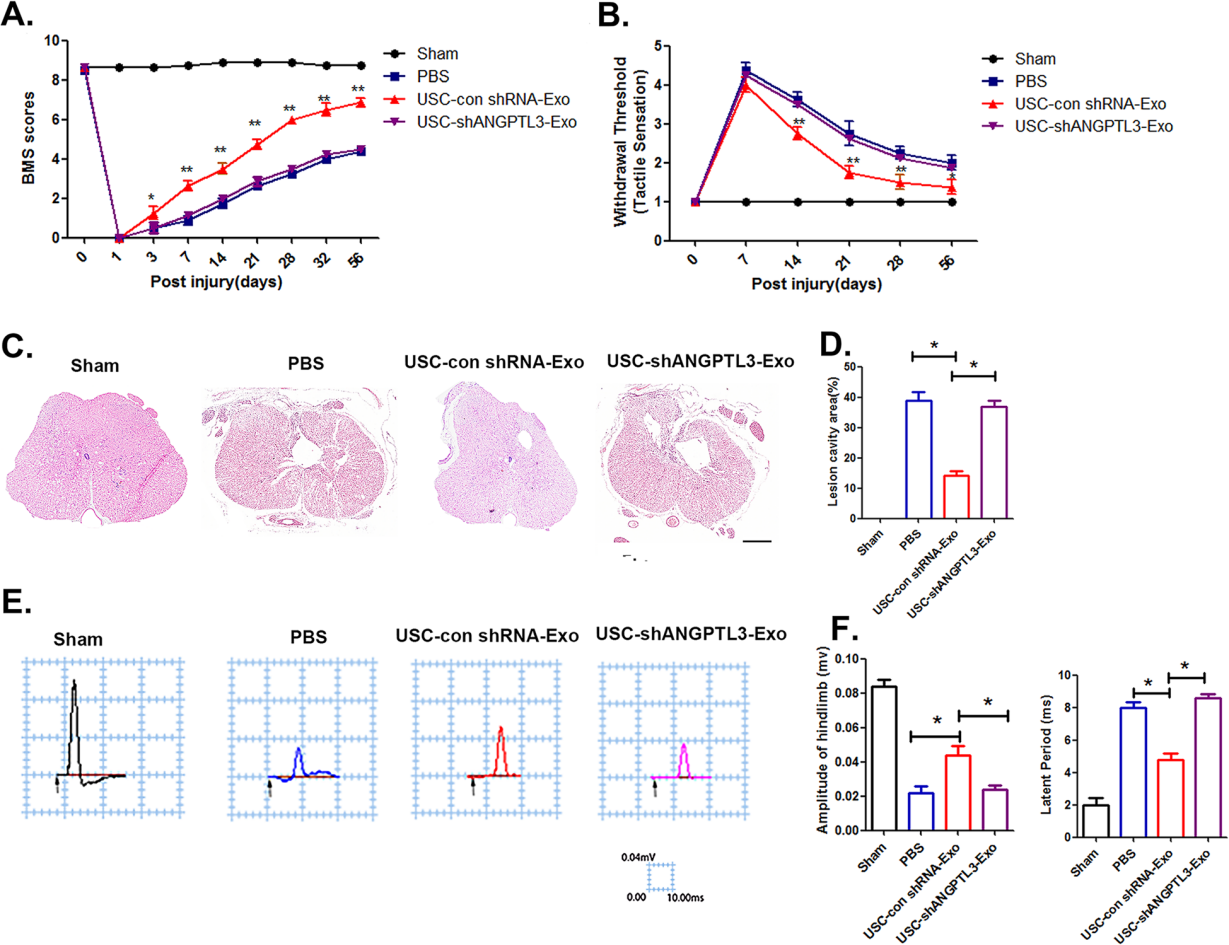
ANGPTL3 present in USC-Exo mediates neurological functional recovery after SCI. **a**, **b** Distribution of BMS scores and withdrawal threshold per group throughout the 56 days. **c** Cross-sectional image of HE staining in the PBS-, USC-Con shRNA-Exo-, and USC-shANGPTL3-Exo-treated mice. Scale bar = 100 μm. **d** Quantitative data of the lesion cavities shown in **c**. **e**, **f** Representative electrophysiological traces and the quantitative data of amplitudes, latent period in the PBS-, USC-Con shRNA-Exo-, and USC-shANGPTL3-Exo-treated mice. The data are presented as the means ± SD; *n* = 6 per group. *p < 0.05, **p < 0.01 compared among different treatment groups

To elucidate the effects of USC-Exo on neurological functional healing through transfer of ANGPTL3 to the injured spinal cord area, we evaluated cargo transfer to the spinal cord injury site and observed a significant increase in ANGPTL3 expression in injured spinal cord tissues at 3 days post-SCI after USC-Exo administration (Supplementary Figure 2). To assess whether the administration of ANGPTL3 carried by exosomes may have an advantage over the use of human recombinant ANGPTL3 (rANGPTL3), we compared their neurological protection effects on spinal cord functional recovery. We observed that mice treated with USC-Exo containing ANGPTL3 at 1 pg/2.0 × 10^8^ exosome particles had a protective effect on neurological functional recovery after SCI. However, treatment with human recombinant ANGPTL3 at the same quantity (1 pg/mouse) did not exert a neurological protective effect in injured spinal cord tissue, as evaluated by motor-evoked potential (MEP) recording (Supplementary Figure 3). To further evaluate the role of ANGPTL3 as the key molecule mediating the neurological protective effect of USC-Exo, we compared the protective effects of exosomes isolated from USC-shANGPTL3-Exo and USC-Con shRNA-Exo. Locomotor BMS score analysis results revealed that USC-Con shRNA-Exo treatment led to significant functional recovery compared to PBS treatment. Specifically, at 28 days post-SCI, the BMS score in the USC-Con shRNA-Exo groups reached 8 ± 0.53, significantly higher than the score of 3.25 ± 0.46 observed in the PBS treatment groups. However, the protective effect of the USC-Exo treatment on functional recovery was markedly decreased with the downregulation of ANGPTL3 expression (Fig. 7a). The withdrawal thresholds in response to mechanical stimuli were assessed to determine the sensory recovery after SCI with the administration of USC-Exo. The data revealed that mice in the USC-Con shRNA-Exo treatment groups attained sensory recovery at 14 days and lasted until 56 days post-SCI. In contrast, the PBS- and USC-shANGPTL3 Exo-treated groups did not show sensory recovery at the same days post-SCI, indicating complete abolishment of the sensory function due to the loss of ANGPTL3 in USC-Exo (Fig. 7b). In addition, histological evaluation of spinal cord sections confirmed the beneficial effect of ANGPTL3 in mediating the protective effect of USC-Con shRNA-Exo on the maintenance of spinal cord tissue morphology, with a limited lesion area observed compared to that observed in the USC-sh-ANGPTL3-Exo-treated mice (Fig. 7c, d). MEP recordings demonstrated that the USC-Con shRNA-Exo treatment group showed a higher amplitude of MEPs and a lower latent period value than the PB-treated mice at 56 days post-SCI, whereas USC-shANGPTL3-Exo with decreased ANGPTL3 levels were completely ineffective in restoring spinal cord neurological function after SCI (Fig. 7e, f). In addition, the expression of regeneration-associated genes (RAGs; ChAT, Hoxd10, and Lhx5) in the injured spinal cord tissue following USC-Con shRNA-Exo treatment was upregulated relative to the control groups at 7 days post-injury. No effect was observed in the USC-shANGPTL3-Exo groups (Supplementary Figure 4). These data suggest that ANGPTL3 present in USC-Exo is responsible for generating a regenerative microenvironment that is associated with improved neurological functional recovery after SCI.

### ANGPTL3 mediates the angiogenesis of USC-Exo in the injured spinal cord after SCI

**Fig. 8:**
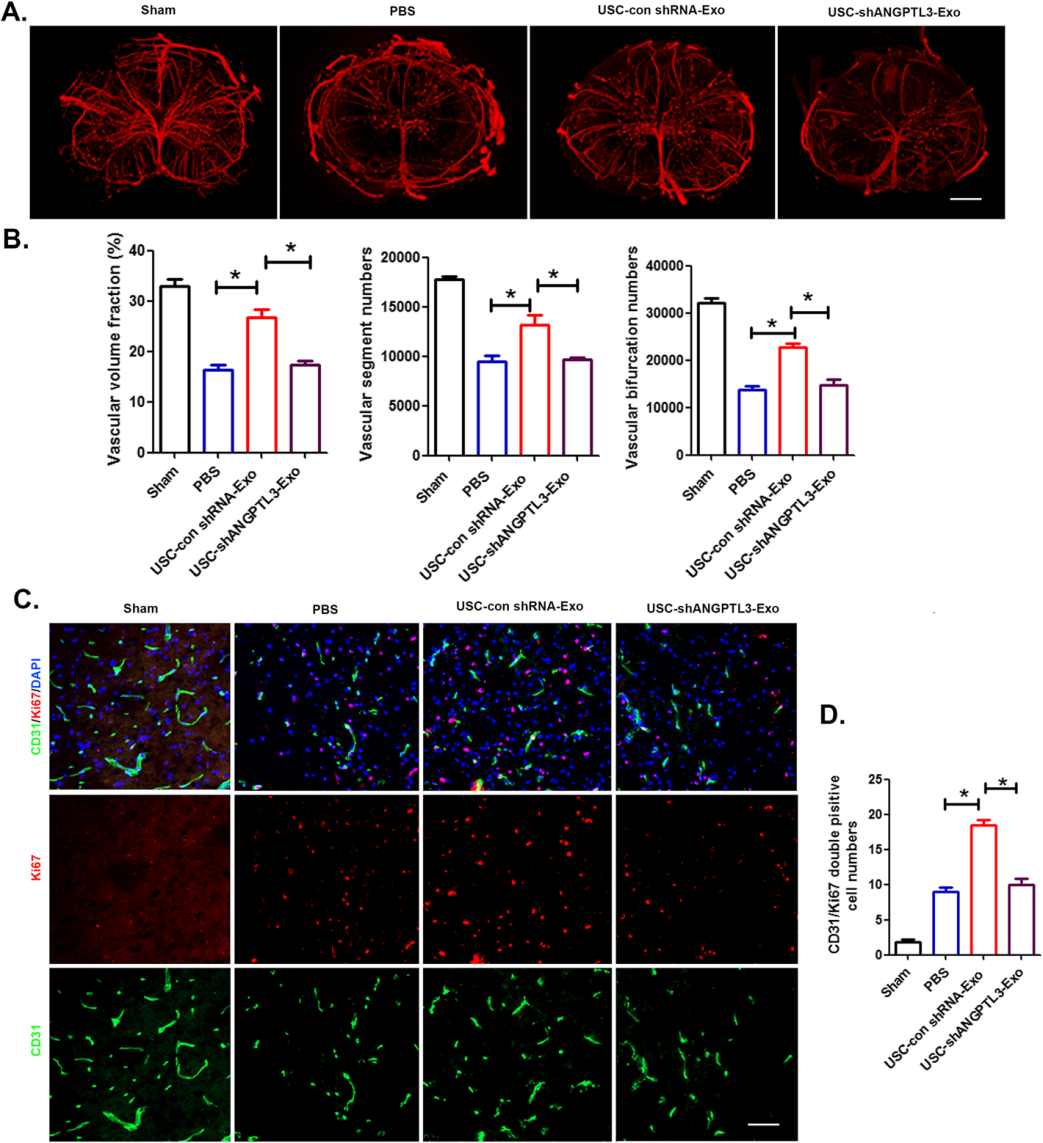
Exosomal ANGPTL3 in USC-Exo promotes angiogenesis at the spinal cord injury site. **a** Gross 3D view of spinal cord microvasculature in the injured spinal cord treated with PBS, USC-Con shRNA-Exo, and USC shANGPTL3 post-SCI; scale bar, 200 μm. **b** Quantitative analysis of the vascular morphological parameters shown in **a**. **c** Representative images of ki67/CD31 double staining of injured spinal cord sections treated with PBS, USC-Con shRNA-Exo, and USC shANGPTL3 post-SCI; scale bar, 50 μm. **d** Quantification of the number of ki67/CD31 double-positive cells shown in **c**. The data are presented as the means ± SD. *p < 0.05, **p < 0.01 compared among different treatment groups

To further assess the effect of exosomal ANGPTL3 on vessel regeneration in the injured spinal cord after SCI, 3D vessel imaging was performed by SRμCT. The results demonstrated that the USC-Con shRNA-Exo treatment promoted new blood vessel formation in mice, as characterized by the upregulated vessel volume fraction, vessel segment, and bifurcation numbers of the spinal cord compared with the control mice treated with PBS at day 28 post-SCI, whereas the pro-angiogenic effect of USC-sh ANGPTL3-Exo was lower than that observed in the USC-Con shRNA-Exo group (Fig. 8a, b). Furthermore, endothelial proliferation of mouse spinal cord vessels in different treatment groups was assessed by double immunostaining for Ki67 and CD31 at day 7 after SCI (Fig. 8c). The immunostaining data demonstrated that compared to the PBS-treated mice, the acceleration of endothelial proliferation was upregulated with increased numbers of Ki67-positive and CD31-positive cells observed in USC-Con shRNA-Exo-treated mice. However, blood vessel formation was attenuated in the USC-sh ANGPTL3-Exo-treated mice (Fig. 8d). These data indicated that ANGPTL3 expression in USC-Exo was responsible for promoting revascularization in the injured spinal cord after SCI and provides a new therapeutic approach for spinal cord injury tissue regeneration.

## Discussion

**Fig. 9:**
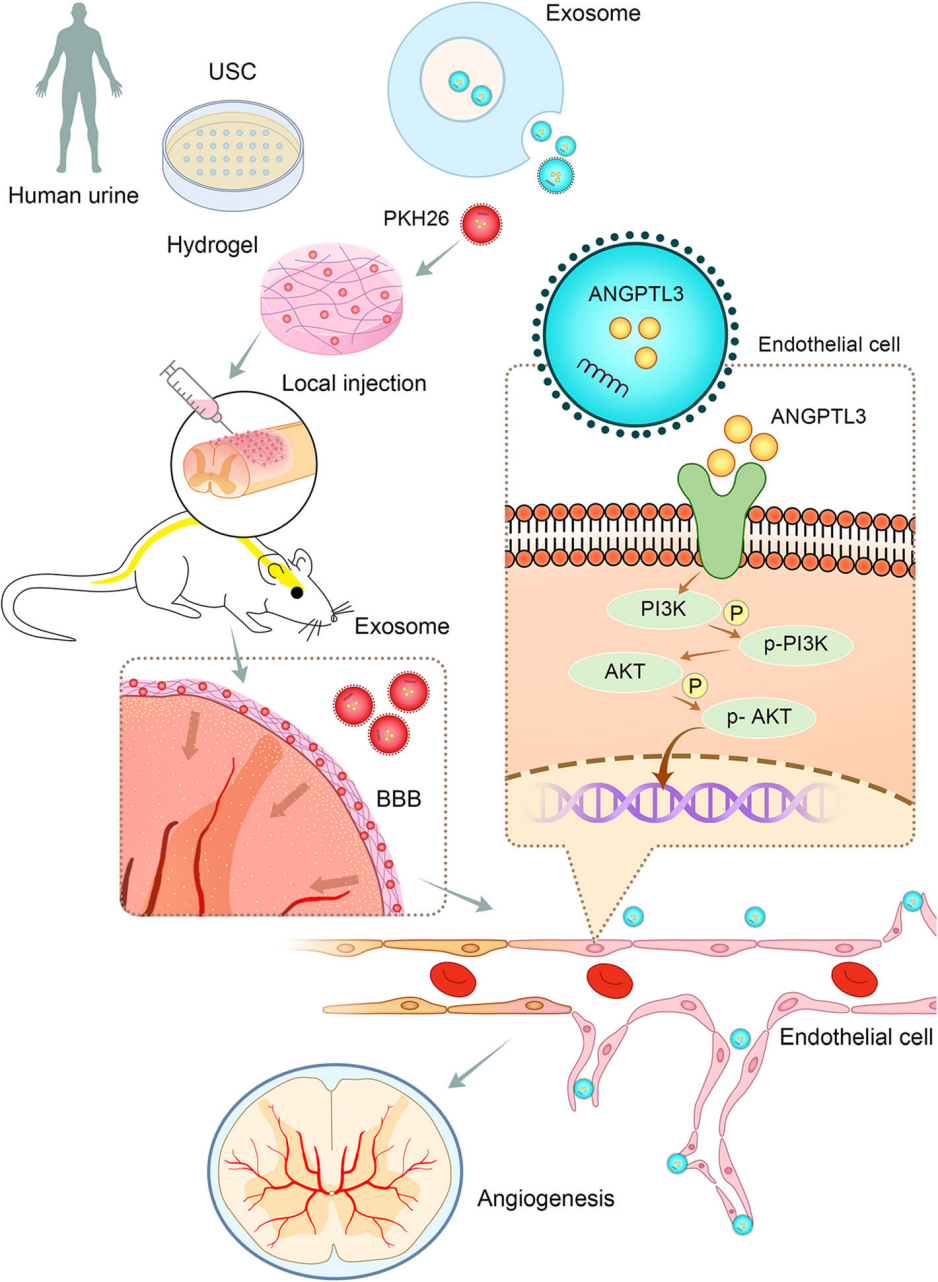
Schematic diagram showing the role of exosomal ANGPTL3 from human USC-derived exosomes in mediating the protective effect on functional recovery after spinal cord injury by promoting angiogenesis. USC-derived exosomes enriched with ANGPTL3 and are imbedded in hydrogel and used to cover the injured spinal cord surface. The exosomes can cross the spinal cord blood-brain barrier (BBB) and be taken up by spinal cord microvasculature endothelial cells, where ANGPTL3 induces PI3K and AKT phosphorylation and subsequently promotes angiogenesis

The limited regenerative capacity of neural tissue inhibits the restoration of normal function in SCI patients. Currently, numerous therapeutic strategies have been developed to enhance spinal cord regeneration after SCI, but unfortunately, none of these approaches has been convincingly efficacious at improving neurologic function in SCI patients to date. To the best of our knowledge, this is the first study to evaluate the functional role of exosomes derived from the USC of healthy subjects in spinal cord regeneration using an experimental model of spinal cord injury with the local delivery of exosomes. Our results demonstrated the neurological protective effect of USC-Exo on spinal cord functional recovery. Specifically, we found that USC-Exo treatment could promote angiogenesis and injured spinal cord healing after SCI in the presence of ANGPTL3 within the exosomes (Fig. 9). However, the beneficial effect of new vessel formation and spinal cord functional recovery after SCI was attenuated with the inhibition of ANGPTL3 expression in USC-Exo. To elucidate the potential mechanism associated with this activity, our in vitro results revealed that USC-Exo treatment could remarkably promote angiogenic tube formation in endothelial cells after they were internalized by HUVECs by activating the PI3K/AKT signaling pathway. Furthermore, vessel tube formation assay results revealed that ANGPTL3 is required for USC-Exo to promote the angiogenic responses in HUVECs. These data indicated that the ANGPTL3 protein may be the key cargo mediating the protective effect of USC-Exo by promoting angiogenesis and injured spinal cord healing.

It is well known that when the spinal cord is injured, local mechanical forces will disrupt the vascular architecture of the cord. The disruption of local vasculature reduces perfusion of the remaining spinal cord parenchyma and leads to neurological deterioration of SCI, highlighting the importance of novel therapies that promote angiogenesis to maximally improve local blood supply to the spinal cord parenchyma, which in turn reduces neurological deficits. Among the range of therapeutic approaches under investigation, the use of exosomes derived from MSCs is considered the most promising therapeutic strategy for spinal cord regeneration [18]. Wei et al. used exosomes derived from BMSCs for SCI treatment and confirmed that they could repair the injured spinal cord by suppressing the activation of A1 neurotoxic reactive astrocytes after traumatic SCI [19]. A study conducted by Sun and colleagues showed that exosomes derived from umbilical cord MSCs exhibit neuroprotective effects and enhance spinal cord functional recovery by reducing inflammation [20]. Taken together, the results of these studies indicated that exosomes derived from MSCs created a favorable microenvironment for spinal cord injury repair. In our present study, we observed that human urine stem cell-derived exosomes could enhance spinal cord functional recovery after injury and remarkedly increase the number of newly formed blood vessels in the injured spinal cord. In vitro tubule formation assay results revealed that USC-Exo could promote the angiogenic activities of HUVECs. These results suggest that the protective effects of USC-Exo in enhancing spinal cord neurological functional healing are likely attributable to its stimulatory effects on endothelial angiogenesis. Compared to BMSCs and umbilical cord MSCs, in our study, the use of exosomes derived from human urine stem cells that could be obtained through a noninvasive approach may be the best cell source for exosome extraction and is considered to be a new innovative therapeutic strategy for spinal cord regeneration in translational clinical settings [21].

The effects of MSC-derived exosomes have been primarily attributed to their cargo molecules, such as proteins, lipids, DNA, and multiple RNA species [22–25]. In the present study, we demonstrated that labeled exosomes could cross the blood-brain barrier (BBB) in the spine and migrate to the injured area. Furthermore, these exosomes could transfer their specific cargo to damaged tissue, including the pro-angiogenic molecule ANGPTL3.

ANGPTL3, a member of the angiopoietin-like (ANGPTL) protein family, is a secreted glycoprotein that has a similar structure to angiopoietin family proteins [26]. ANGPTLs are widely expressed in the vascular system and play important roles in angiogenesis [27]. To date, eight ANGPTLs (ANGPTL1 to ANGPTL8) have been discovered [11]. ANGPTL3 is functionally defined by the C-terminal fibrinogen-like domain that mediates binding to the Tie2 receptor and has been shown to be involved in new blood vessel formation [28]. ANGPLT3 was reported to bind to integrin ανβ3 and simulate endothelial cell migration, indicating a possible role of ANGPLT3 in regulating angiogenesis [29]. In addition, the administration of ANGPTL3 has been shown to stimulate the phosphorylation of AKT in HUVECs, which contributes to its pro-angiogenic ability [26].

Similarly, in our present study, we observed that ANGPTL3 was highly expressed in USC-derived exosomes and could be transferred to the SCI site to promote spinal cord repair. However, the effect of recombinant ANGPTL3 administered alone did not exert any regenerative effect on SCI functional recovery. This result further emphasizes the observation that exosomes can function as novel biocarriers to create synergistic effects with the molecules they harbor.

In addition, exosomes are a cell-free treatment option that are nonimmunogenic in nature, possess an intrinsic ability to be selectively delivered to target tissues, and have long circulation times in the body, making them ideal candidates for targeted therapy by effectively transferring different types of molecules upon genetic modification [30–32]. Biological modification with pro-regenerative factors may enhance the innate therapeutic capability of exosomes [33]. In addition, due to their native structure and characteristics, exosomes function as promising slow-release reservoirs of molecules inside the body, providing a steady stream of the therapeutic molecules [18, 30, 34]. Guo et al. tested experimental SCI models loaded with PTEN-siRNA-MSC-Exos and showed that they could efficiently carry PTEN-siRNA to the target tissues and augment regenerative effects by promoting more robust axon regeneration after SCI [35].

In our present study, we developed an engineered exosome treatment based on reprogramming exosomes to harbor therapeutic proteins that can be edited. The expression of ANGPTL3 in exosomes was downregulated using siRNA technology. Previous studies also showed that fusing the exosomal membrane protein Lamp2b with a brain-specific rabies virus glycoprotein (RVG) peptide could increase the targeting capacity of exosomes and allow them to cross the BBB [36, 37]. Thus, future exosome research will involve fusing the highly expressed ANGPTL3 exosome derived from USCs with RVG to enhance the delivery of cargo across the BBB to efficiently reach the target tissue. Thus, compared with naïve USC-derived exosomes, engineered USC-derived exosomes may have more potent therapeutic effects in SCI.

The promotion of angiogenesis involves the activation of multiple signaling pathways. It has also been reported that activation of the phosphatidylinositol 3-kinase (PI3K)/AKT pathway is known to regulate multiple crucial steps in angiogenesis, including endothelial cell survival and capillary-like structure formation [38]. Furthermore, the PI3K/AKT signaling pathway also regulates the expression of angiogenic factors such as angiopoietins [39]. Recently, the PI3K/AKT signaling axis was shown to be activated by a variety of stimuli in endothelial cells [40]. The effects of VEGF on endothelial cells have been demonstrated to be mediated by the PI3K/AKT pathway [40]. In our present study, the PI3K/AKT signaling pathway was activated in HUVECs after the administration of USC-Exo, which may be attributed to the high expression levels of the pro-angiogenic factor ANGPLT3 protein cargo in the exosomes. Future work will be required to elucidate the exact underlying mechanism by which exosomal ANGPLT3 derived from USC activates PI3K/AKT signaling in endothelial cells to promote angiogenesis. While the effect of USC-Exo on angiogenesis after spinal cord injury was observed in the present study, the limitations of this study should be considered when interpreting the results. Following administration, DiR-labeled exosomes were observed to be taken up by endothelial cells. However, the labeled exosomes also colocalized with other types of cells in the SCI sites. Thus, future studies should examine the effects of USC-Exo on neurogenesis to determine whether our findings are vessel specific.

## Conclusions

In summary, the results of the present indicate a novel therapeutic strategy for spinal cord regeneration. Exosomes derived from the USC of healthy subjects appear to be a novel source of exosomes with pro-regenerative properties. Our findings revealed that the local delivery of USC-Exo may enhance spinal cord neurological functional recovery by creating a regenerative environment that promotes angiogenesis by transferring the pro-angiogenic factor ANGPLT3 protein. This effective cell-free treatment holds great promise for future clinical translation in SCI patients.

## Supplementary Information

**Additional file 1.**

## Data Availability

All data generated or analyzed during this study are included in this published article.

## Abbreviations

SCI: Spinal cord injury
ANGPLT3: Angiopoietin-like protein 3
HUVEC: Human umbilical vein endothelial cell
USC: Urine stem cell
Exo: Exosome
NTA: Nanoparticle tracking analysis
TEM: Transmission electron microscope
WB: Western blot
MEPs: Motor-evoked potentials
SRμCT: Synchrotron radiation micro-CT
SSRF: Shanghai Synchrotron Radiation Facility
VVF: Vessel volume fraction
VSN: Vessel segment number
VBN: Vessel bifurcation number
RVG: Rabies virus glycoprotein
BBB: Blood-brain barrier
