# Supplementary material for: Local delivery of USC-derived exosomes harboring ANGPTL3 enhances spinal cord functional recovery after injury by promoting angiogenesis
Source: Stem Cell Res Ther. 2021 Jan 7;12:20. doi: 10.1186/s13287-020-02078-8 (PMC7791988; doi:10.1186/s13287-020-02078-8)
Supplement: Supplementary file 1 — Additional file 1. [file 13287_2020_2078_MOESM1_ESM.docx]

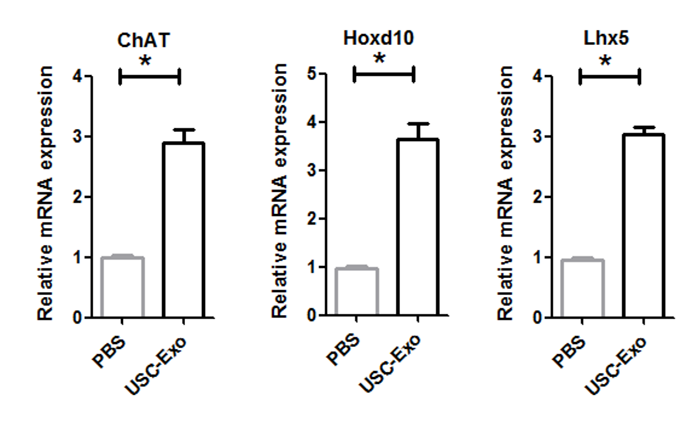


**Supplementary figure1.** USC-Exo promote the regeneration associate genes expression. Regeneration associated genes ChAT, Hoxd10 Lhx5 were analysis by qRT-PCR. Data present as mean ± SD, *P < 0.05, compared with PBS group


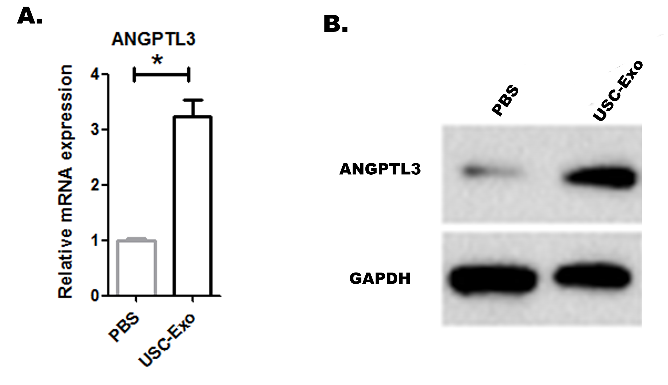


**Supplementary figure2.** The ANGPTL3 expression after the treatment with USC-Exo. (A) The mRNA expression of ANGPTL3 analysis by qRT-PCR. (B) The protein level of ANGPTL3 investigated via Western blot. Data present as mean ± SD, *P < 0.05, compared with PBS group.


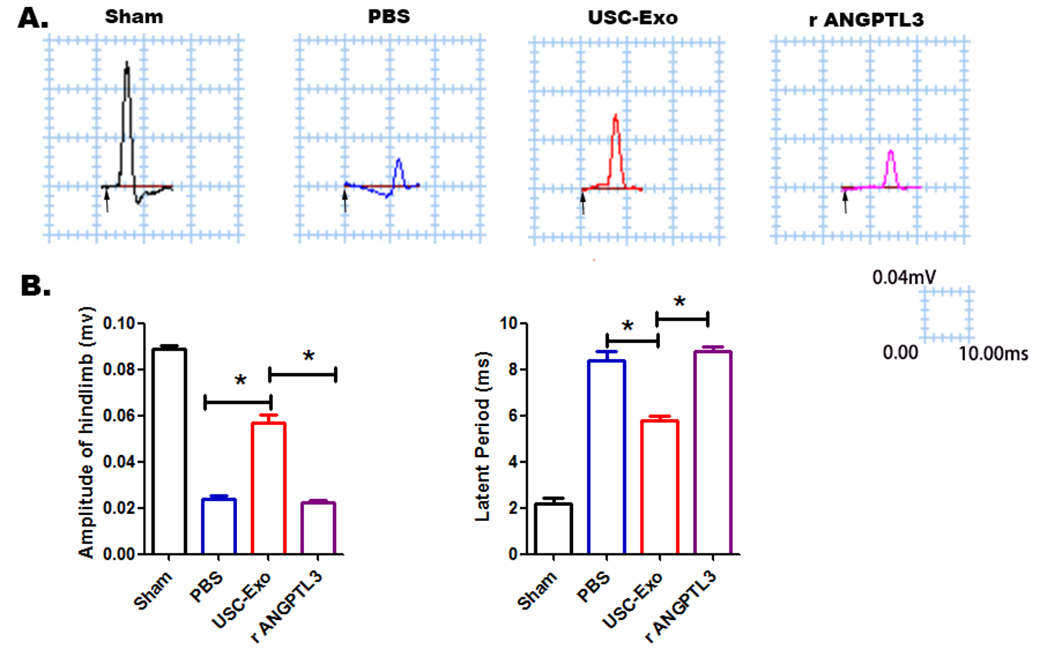


**Supplementary figure 3.** The hindlimb locomotor function was assessed using Motor evoked potentials (MEP) at 56 days after SCI. (A) Representative electrophysiological traces in sham,

PBS, USC-Exo and r ANGPTL3- treated mice. (B) The quantitative data of amplitude and latent period in different treatment groups. Data present as mean ± SD, *P < 0.05, compared among different treatment group.


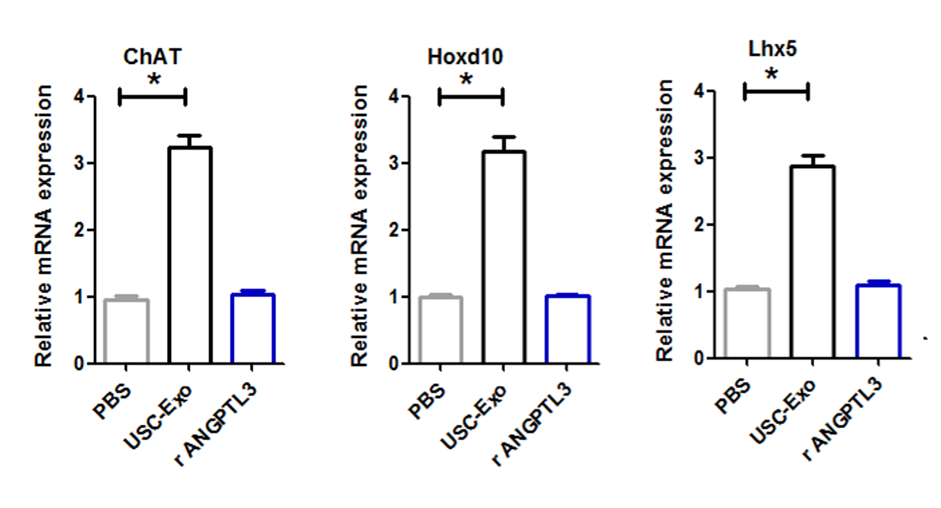


**Supplementary figure4.** The regeneration associate genes expression in PBS, USC-Exo and r ANGPTL3 treated groups. The gene of ChAT, Hoxd10 Lhx5 expression were analysis by qRT-PCR. Data present as mean ± SD, *P < 0.05, compared with PBS group

**Supplementary Table1** Primer sequence for qRT-PCR

| Gene | Forward (5′-3′) | Reverse (5′-3′) |
| --- | --- | --- |
| ANGPTL3 | CACTTCAACTGTCCAGAGGGTTA | GTTTTCTCCACACTCATCATGC |
| GAPDH | GTCTCCTCTGACTTCAACAGCG | ACCACCCTGTTGCTGTAGCCAA |
| ChaT | GGCTTTTGTGCAAGCCATGA | CACAGGGCCATAACAGCAGA |
| Hoxd10 | TGTACAGTGCAGAGAAGCGG | GTGTCTGGACTGGAGTCTGC |
| Lhx5 | CAGGATCCGTTACAGGACGA | AACCACACCTGAATGACCCT |
